# Sucrose reduction with maintained sweetness level lowers glycemic fluctuations and energy intake in healthy males

**DOI:** 10.3389/fnut.2025.1682297

**Published:** 2025-11-03

**Authors:** Marlies Gaider, Isabella Kimmeswenger, Jana Schmidt, Cynthia Thines, Anni Wu, Teresa K. Stoffl, Petra Rust, Jakob P. Ley, Gerhard E. Krammer, Veronika Somoza, Barbara Lieder

**Affiliations:** ^1^Christian Doppler Laboratory for Taste Research, Faculty of Chemistry, University of Vienna, Vienna, Austria; ^2^Vienna Doctoral School of Chemistry (DoSChem), University of Vienna, Vienna, Austria; ^3^Department Human Nutrition and Dietetics, Institute of Clinical Nutrition, University of Hohenheim, Stuttgart, Germany; ^4^Institute of Physiological Chemistry, Faculty of Chemistry, University of Vienna, Vienna, Vienna, Austria; ^5^Department of Nutrional Science, Faculty of Life Sciences, University of Vienna, Vienna, Austria; ^6^Symrise AG, Holzminden, Germany

**Keywords:** appetite, blood glucose, energy intake, sweet taste modulation, sweet taste, sucrose

## Abstract

**Introduction:**

The sole perception of sweet taste is discussed to interfere with postprandial blood glucose regulation and leading to enhanced cravings for sweet foods. This raises the question whether preserving sweetness while reducing sugar in a test solution can sustain beneficial effects on blood glucose regulation and subsequently decrease postprandial energy intake. Specifically, we hypothesized that reducing the caloric load of a sucrose solution while maintaining the perceived sweetness intensity by adding hesperetin as a taste modifier attenuates large fluctuations in postprandial blood glucose concentrations with beneficial effects on appetite and cravings for sweet foods.

**Methods:**

In a randomized crossover study with 32 healthy male participants, the effect of a 10% sucrose solution on blood glucose regulation and energy intake was compared to an equi-sweet 7% sucrose solution with 50 mg/L hesperetin. Data was analyzed using paired Student’s t-tests or Repeated-measures ANOVA. The study was approved by the ethical committee of the University of Vienna (approval number 00903) and registered at ClinicalTrials.gov (NCT05705596).

**Results:**

The results show that the decline in blood glucose concentrations was less pronounced after consumption of the 7% sucrose solution with hesperetin than after the isosweet 10% sucrose solution. Additionally, participants reported less desire for a sweet snack and had on average a 10 ± 7% (*p* < 0.05) lower energy intake after consumption of the 7% sucrose hesperetin-spiked solution.

**Conclusion:**

In conclusion, our results argue for a pronounced role of the carbohydrate content in postprandial appetite regulation.

## Graphical abstract

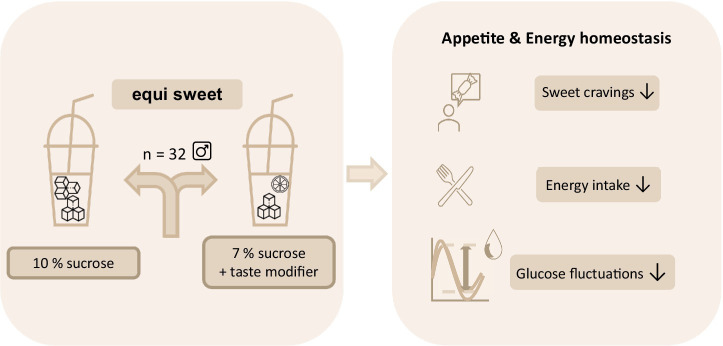

## 1 Introduction

Taste is considered to be one of the key drivers for the decision to consume foods. Among the primary taste modalities, sweetness is consistently identified as the most highly preferred sensory attribute (1). Sweet taste is mediated by the canonical sweet taste receptor, consisting of the subunits TAS1R2 and TAS1R3 (2). Besides the oral cavity, presence of the sweet taste receptor has also been proven in several non-gustatory tissues, such as the gastrointestinal tract. Here, the TAS1R3 subunit has been found to be the predominant form. Stimulation of extraoral TAS1R3 has been shown to be involved in the expression and secretion of gastrointestinal appetite-regulating hormones such as glucagon-like peptide-1(GLP-1) (3), gastric inhibitory peptide (GIP) (4) and the neurotransmitter serotonin (3). The activation of TAS1R3 also stimulates the release of several peptide hormones in enteroendocrine cells, including leptin, ghrelin, peptide tyrosine-tyrosine (PYY) and cholecystokinin (5). Some of these anorectic hormones, e.g., GLP-1, GIP and PYY, get rapidly degraded by the enzyme Dipeptidyl-peptidase 4 (DPP4) (6, 7). Therefore, DPP4 is a key player not only in glucose homeostasis, but also important for the regulation of food intake and DPP4-inhibitors provide a promising target for regulating blood glucose fluctuations and insulin sensitivity in type two diabetes mellitus treatment (8). In addition, the activity of DPP4 has been recently shown to be regulated by TAS1R3 activation in enterocytes (9). Those studies demonstrate a prominent role for TAS1R3 for appetite regulation on a mechanistic level. Moreover, besides the activation of extraoral sweet taste receptor subunits, the impact of the sole perception of sweetness in the regulation of metabolic functions has been controversially discussed in literature. Particular emphasis has been directed towards cephalic phase insulin release and its role in postprandial hyperglycemia, as recently reviewed by Langhans et al. (10). Although findings in isolated islets supported a role for the sweet taste receptor subunits in insulin release (11), results from *in vivo* studies were more controversial. While Just and colleagues showed a stimulation of insulin release following the tasting of a sucrose solution and spitting it out afterwards (12), other studies did not detect increased insulin secretion by just tasting the disaccharide without swallowing (13–15). In those studies, however, accidental swallowing of small amounts of sucrose cannot be completely excluded. Previous studies of our own group however demonstrated that the intensity of sweetness perception had no effect on blood glucose regulation after administration of isocaloric glucose or sucrose-containing solutions in combination with the sweet taste receptor antagonist lactisole (16). Similarly, modulation of the sweetness level of a sucrose solution by addition of lactisole or rebaudioside M did not affect postprandial blood glucose regulation and energy intake regardless of the sweet taste sensitivity of the test persons (17). However, the addition of lactisole to a sucrose, but not glucose solution reduced postprandial energy intake (18) and GLP-1 plasma concentrations (16), arguing for an interplay of glucose transporters and the sweet taste receptor for nutrient sensing. Therefore, it remains unclear whether reducing the sucrose load while maintaining the perceived sweetness intensity will have beneficial effects expected of a reduced sugar content, or whether the similar activation of oral and/or extra-oral sweet taste receptors will lead to a similar hormonal response as the higher concentrated sugar solution. Maintaining sweetness while reducing sugar content can be achieved using flavoring substances with modifying properties (FMP). FMPs have no or a low intrinsic sweet taste but can modify the perceived sweetness in combination with sugar. This allows for sugar reduction while preserving the consumer’s preferred sweet taste without the undesired side tastes of other non-nutritive sweetener (19).

Based on our previous findings, we hypothesized that the caloric load of a sugar-sweetened beverage is more important for the blood glucose regulation than the perceived sweetness and hence, the reduction of sugar while maintaining the sweetness has positive effects on the postprandial blood glucose response and subsequent cravings. The naturally derived flavoring substance hesperetin was used in the present study as a taste modifier. Hesperetin is known for flavor modulating properties with very limited intrinsic sweet taste *in vivo* (20). In combination with sucrose it exhibits synergistic effects on the sweetness intensity (21) and consequently has taste modulating properties. Previous studies demonstrated that hesperetin can activate the sweet receptor *in vitro* starting from concentrations of 0.025 mM (22) and exhibit bitter-masking effects (23). Hesperetin represents the aglyconic form of the flavanone hesperidin. In the human intestine hesperetin is absorbed after the removal of rutinose or via hesperetin 7-glucoside from hesperidin by bacterial enzymes, followed by conversion to glucuronidated and sulfated metabolites, and ultimately excreted in the urine or bile (24). Safety assessments of the glycoside hesperidin and related substances identified no adverse effects from oral exposure of up to 1,000 mg/day of hesperidin (25, 26). The flavoring substance hesperetin is classified as generally recognized as safe (GRAS) by the Flavors and Extracts Manufacturing Association (FEMA) and approved as flavoring substance for human consumption by the European Food Safety Authority (EFSA) (27).

Using the synergistic combined sweet intensity of sucrose and hesperetin, this study investigated the short-term metabolic effects of reducing the sugar content of a 10% sucrose solution while maintaining the perceived sweet taste by adding 50 mg/L of the flavoring substance hesperetin to a 7% sucrose solution in healthy male volunteers in a randomized cross-over design.

## 2 Materials and methods

### 2.1 Participants

**Figure 1:**
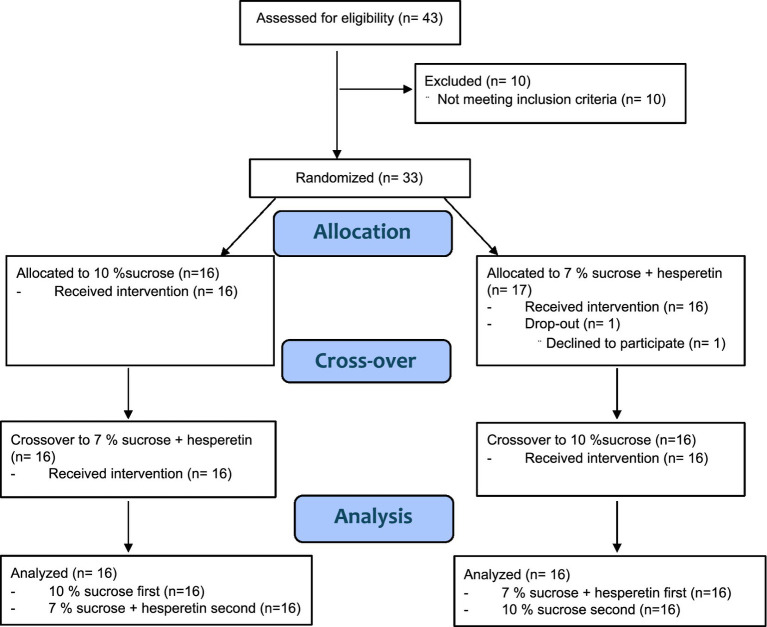
CONSORT flow diagram.

Power analysis by means of the software G-Power 3.1 (28) resulted in an estimated number of 31–35 participants, based on differences of blood sugar peaks in previous studies (16, 29) with an effect size of 0.5 (29) or 0.43 (16) (power of 0.85, alpha = 0.05). Forty-three males were recruited to participate in a medical screening by advertisements on social media and postings at the University of Vienna. Eligible participants were metabolically healthy males between 18 and 45 years old with a Body Mass Index (BMI) between 18.5–29.9 kg/m^2^ and no self-reported taste or odor dysfunction. Additionally, participants were required to have a body fat percentage below 30% to ensure inclusion of non-obese subjects, recognizing that also individuals with a normal BMI may still exhibit metabolic dysfunctions associated with increased visceral adipose tissue (summarized by Ding et al. (30)). Exclusion criteria were adopted from previous studies and are described in detail elsewhere (17). Females were excluded to avoid interference with fluctuations of blood glucose following hormonal variances during menstrual cycle (31). Furthermore, participants were required to have a sucrose recognition threshold < 12.1 g/L as previous research has shown that sweet taste perception is associated with postprandial glucose regulation (17) and energy intake (32). Lastly, to assess general preference for sweet foods and beverages participants completed a sweet preference questionnaire. In total, 33 participants fulfilled all criteria, and 32 completed all intervention days. One person withdrew from the study due to personal reasons (Figure 1). All participants provided written informed consent in accordance with the Declaration of Helsinki prior to the study. The study was approved by the ethical committee of the University of Vienna (approval no. 00903) and registered at ClinicalTrials.gov (NCT05705596).

### 2.2 Study design

The interventional part of the study was carried out between January 2023 and May 2023 as a single blinded, randomized, cross-over, human intervention study at the facilities of the Institute for Physiological Chemistry at the University of Vienna with two treatments: 10% (w/v) sucrose in 300 ml of water and 7% (w/v) sucrose with 50 mg/L hesperetin. Participants received one intervention per study day, with at least 5 days between the interventions and were blinded about the order of the treatments (single-blinded study design). The order of the interventions was allocated using the online randomizer “randomizer.org.” The primary outcomes were the time-dependent fluctuations in blood glucose concentrations and the 2 h post-load *ad libitum* energy intake. To investigate the underlying mechanisms, the individual appetite score, metabolic and hormonal responses to the interventions were measured as secondary outcome parameters.

### 2.3 Test solutions

Given the typical sugar content of soft drinks, we selected a sucrose concentration of 10% (w/v) in 300 ml of water, corresponding to 120 kcal. The isosweet solution with a reduced caloric load contained a sucrose concentration of 7% (w/v) in 300 ml of water and 50 mg/L of the flavanoid hesperetin and provided 84 kcal. Based on sensory assessment of trained panelists, the addition of 50 mg/L hesperetin to 7% sucrose was suitable to reach the sweetness level of a 10% sucrose solution (see Supplementary Figure 1).

### 2.4 Procedure

All volunteers were invited for a medical screening involving an oral glucose tolerance test, anthropometric characterizations, blood markers of metabolic and hepatic health. The procedures performed can be found in more detail in a previous study by Preinfalk et al. (17).

The participants that fulfilled all requirements were invited for two consecutive study days. On the study days, the test persons arrived after a 12 h overnight fast and blood samples were collected before and 15, 30, 60, 90, and 120 min after administration of the test solution. Participants were instructed to drink the solution within a time frame of 5 min and asked to rate the sweetness intensity of the respective treatment on a 10 cm horizontally presented, unstructured scale (0 cm = not at all and 10 cm = very intensive). After the last blood collection, a standardized *ad libitum* breakfast with a total energy content of 2,952 kcal was served, and the energy and nutrient intake was analyzed by weighing the leftovers and calculated using the software nut.s (nutritional.software, Vienna, Austria) as described in the “Total energy intake” section.

#### 2.4.1 Determination of sucrose recognition threshold

Sucrose was purchased from local supermarkets in Vienna and dissolved in tap water prior to the sensory evaluations. The determination of participants sucrose recognition threshold was performed by a series of ten three-alternative forced choice (3-AFC) tests. In brief, test persons received ten triplets, consisting of two 20 ml blanks with tap water and one 20 ml sample of sucrose solutions. Samples were provided in 40 ml clear plastic beakers labeled with randomly assigned three-digit codes. Participants were instructed to neutralize with tap water before the test and between the triplets. The ascending concentrations of sucrose dissolved in tap water ranged from 0.34 to 12 g/L in accordance with the DIN EN ISO 3972-2013:12. Participants were asked to identify the one sample differing from water in each triplet and further specify their choice by indicating the differing attribute. All tests were carried out in sip-and-spit mode. The threshold value reported here refers to the sucrose concentration at which the test persons first accurately identified the stimuli as “sweet” and is subsequently used as a measure for sweet sensitivity. The distribution of determined sucrose recognition thresholds can be found in the Supplementary Figure 2A.

#### 2.4.2 Sweet preference questionnaire

To determine the participants’ preference for sweet and sweet-fatty foods, they completed an online questionnaire on the first study day. The questionnaire based on a validated instrument by Deglaire and colleagues (33), was adjusted to the Austrian dietary habits and consisted of four sections:

(1)  Food liking: Participants rated their liking of specific foods on a 9-point scale (from “I do not like it at all” to “I like it very much”).
(2)  Preferred sweetness level in meals: Participants selected their preferred amount of sweet-tasting food in certain meals using images depicting dishes with increasing amounts of sweet foods.
(3)  Drink choices when dining out: Participants chose among sweet and non-sweet beverages what they would order when eating out.
(4)  Sweet-related eating behavior: Participants rated psychological and social aspects of their dietary behavior concerning sweet foods, with responses arranged in ascending order.

Participants were instructed to rate how pleasant they found the foods, regardless of whether they would actually consume them. For parts 1, 2, and 4, numerical values were assigned to the answer options, with the lowest sweetness preference (e.g., “I do not like it at all”) scored as 1, and higher values reflecting stronger preferences (e.g., “I like it very much” = 9). In part 3 participants selected three drinks, each sweet drink scored as 1, and non-sweet drinks as 0. An overall sweet liking score was obtained by summing scores across all four components, where higher scores indicate greater preference for sweet foods. Finally, the resulting raw score was normalized to a 0–100 scale to facilitate comparability.

#### 2.4.3 Plasma preparation for analysis of total glucose, insulin, GLP-1, GIP, PYY, ghrelin, serotonin and DPP4

For the quantification of plasma glucose concentrations, venous blood samples were collected in fluoride-coated monovettes (Sarstedt, Germany), and for insulin heparin-coated monovettes (Sarstedt, Germany) were used. To determine plasma concentrations of GLP-1, GIP, PYY, ghrelin, serotonin and DPP4 activity, blood was collected in EDTA-coated monovettes. Plasma samples were prepared as described previously (16–18).

#### 2.4.4 Plasma concentrations of total glucose, insulin, GLP-1, GIP, PYY, ghrelin and serotonin

Plasma glucose was quantified using a colorimetric assay (Cayman Europe, Estonia). Insulin (Biorbyt Ltd., UK), GLP-1 (Merck Millipore, Germany), GIP (Ray Biotech, USA), PYY (Merck Millipore, Germany) and ghrelin (Merck Millipore, Germany) levels in the plasma were analyzed using sandwich ELISA according to the manufacturer’s protocol, respectively. Plasma concentration of serotonin was assessed by competitive ELISA (DLD Diagnostic, Germany).

#### 2.4.5 Plasma activity of DPP4

DPP4 enzyme activity was analyzed using a fluorogenic in-house method. The DPP4 activity was determined by the rate of AMC (7-Amino-4-methylcoumarin) cleavage from the DPP4 substrate (Gly-Pro-7-amido-4-methylcoumarin hydrobromide, Sigma-Aldrich, USA), whereby one Unit of DPP4 activity is defined as the amount of DPP4 that hydrolyses the substrate to yield 1 μmol AMC per minute at 37 °C. Human plasma samples were diluted in 20 mM Tris–HCl Buffer (pH 8.0, containing 100 mM NaCl and 1 mM EDTA). For a background control, Sitagliptin (Sigma-Aldrich, USA) was added to the diluted samples reaching a final concentration of 10 μM in 100 μl. A volume of 60 μl of each diluted sample and the respective background sample was pipetted in duplicates on a 96-well plate and incubated at 37 °C for 30 min. To initiate the enzyme reaction, 40 μl DPP4 substrate, diluted in Tris Buffer, was added in a final concentration of 0.3 mM per well. The generated fluorescent cleavage product AMC was measured using a fluorescent microplate reader (Flex Station III, Molecular Devices, Germany) at an excitation wavelength of 360 nm and an emission wavelength of 460 nm in a kinetic mode over 30 min at 37 °C and the concentration determined by comparison to a standard curve. The intra- and intervariabiltiy was 2.45 and 8.64%, respectively.

#### 2.4.6 Appetite score

Participants reported their subjective feeling of hunger, feeling of fullness, desire for a meal and desire for a sweet snack before and 120 min after administering the test solution on four separate visual analogue scales (VAS). The VAS was designed as a digital 10 cm unstructured scale on the online platform *SoSci Survey*, starting at 0 for “not at all” to 10 cm “extremely.” The total appetite score per timepoint was calculated with the following formula, previously described by Markus and Rogers (34):


$$\text{appetite score}=\frac{\left(\begin{array}{l} \text{hunger}+(100-\text{fullness})+\text{desire for}\;a\;\text{meal}\\ +\text{desire for}\;a\;\text{sweet snack} \end{array}\right)}{4}$$

#### 2.4.7 Total energy intake

Following the final blood collection, the total energy intake was determined based on a standardized, continental, *ad libitum* breakfast that is representative of the typical Austrian population. The breakfast, consisting of 48% carbohydrates, 36% fat, and 14% protein, had a total energy content of approximately 2,952 kcal. The breakfast included four bread rolls (~ 260 g), three slices of bread (~ 140 g), 80 g of butter, 60 g of honey, 100 g of strawberry jam, 6 slices of cheese (~ 125 g), 5 slices of ham (~ 100 g), 180 g of fruit yogurt, 200 ml of coffee or tea, 20 g of sugar, 40 g of coffee creamer, and 200 ml of water. Detailed nutritional information of the products used can be found in the Supplementary Table 1. Participants adhering to a vegetarian or vegan diet received calorie-adjusted plant-based alternatives. The quantitative assessment of energy consumption involved back-weighing of unconsumed food, and the software nut.s v1.32.50 (nutritional.software, Vienna, Austria) was utilized for the calculation of energy and nutrient intake.

### 2.5 Statistical analyses

Statistical analyses were performed using GraphPad Prism 10. Data was controlled for normality using Shapiro–Wilk test, and sphericity was assumed and corrected by Greenhous-Geisser method. Difference in baseline corrected hormone concentrations and DPP4 activity over time between treatments was assessed by means of repeated measure two-way ANOVA following Šidák’s Multiple Comparisons Test. Also, appetite ratings were analyzed pre- and 120 min post-intervention by applying a repeated measure two-way ANOVA followed by a Tukey’s Multiple Comparison Test. The baseline corrected net incremental area under the curve (AUC) was calculated with trapezoidal rule. For all analyzed hormones and DPP4 activity, the net incremental ∆-AUC over time was assessed and compared between treatments with a paired two-tailed Student’s t test. The data is depicted as mean ± standard deviation (SD).

For comparisons of the sweetness ratings and energy intake between the treatments paired two-tailed Students-t Test were applied and are presented as mean with individual values depicted as dots.

To determine associations between glucose concentrations and energy intake, sugar intake and appetite, Pearson’s Product Moment correlation analysis was performed.

## 3 Results

In this study, we hypothesized that reducing the caloric load of a sucrose solution while maintaining the perceived sweetness intensity by adding a FMP attenuates large fluctuations in the postprandial blood glucose regulation with beneficial effects on appetite and cravings for sweet foods. This may ultimately reduce postprandial total energy intake. In a randomized cross-over human intervention study, the test persons received two equi-sweet test solutions differing in their caloric load on two separate test days in a randomized order. Our study population consisted of 32 normal-weight adults with an average BMI of 23.5 ± 2.3 kg/m^2^ and a mean body fat percentage of 16.5 ± 6.6%. Supplementary Figures 2B–D illustrate the distribution of body weight, BMI and body fat percentage among the study cohort. Participants also exhibited an average sweet preference score of 71.5 ± 9.4 out of a possible 100 points. All participants reached a sucrose recognition threshold below 12.1 g sucrose per liter and there was no correlation with participants BMI or body fat percentage identified (data not shown). Detailed anthropometric characteristics are presented in Table 1.

**Table 1:** Characteristics of the study participants population.

Characteristics	Mean (min.–max.)
*n*	32
Age [years]	27 (21–42)
Body height [m]	1.81 (1.64–2.05)
Body weight [kg]	77.6 (57.8–97.9)
BMI [kg/m^2^]	23.5 (18.5–28.6)
Body fat [%]	16.54 (5.00–29.90)
Preference sweet food	71.5 (492–87.5)
Sucrose recognition threshold	5.32 (0.55–12)

BMI, Body Mass Index.

### 3.1 Sensorially untrained participants perceived the two treatments as equi-sweet

The equi-sweetness of the test solutions was evaluated by a sensorially trained panel in a preliminary sensory trial. The trained panelists rated the sweetness level on a 100 mm Visual-Analogue-Scale (VAS) of the two test solutions as equal (∆ 2.6 ± 8.5 mm, *p* > 0.05; *n* = 25, Supplementary Figure 1A). This result was verified with the sensorially naïve study participants that reported similar sweetness for both test solutions on the different study days (∆ 6.5 ± 15.5 mm, *p* > 0.05; *n* = 32, Supplementary Figure 1B).

### 3.2 Glucose concentrations showed less fluctuations after consumption of the sugar-reduced solution compared to the full sugar solution

**Figure 2:**
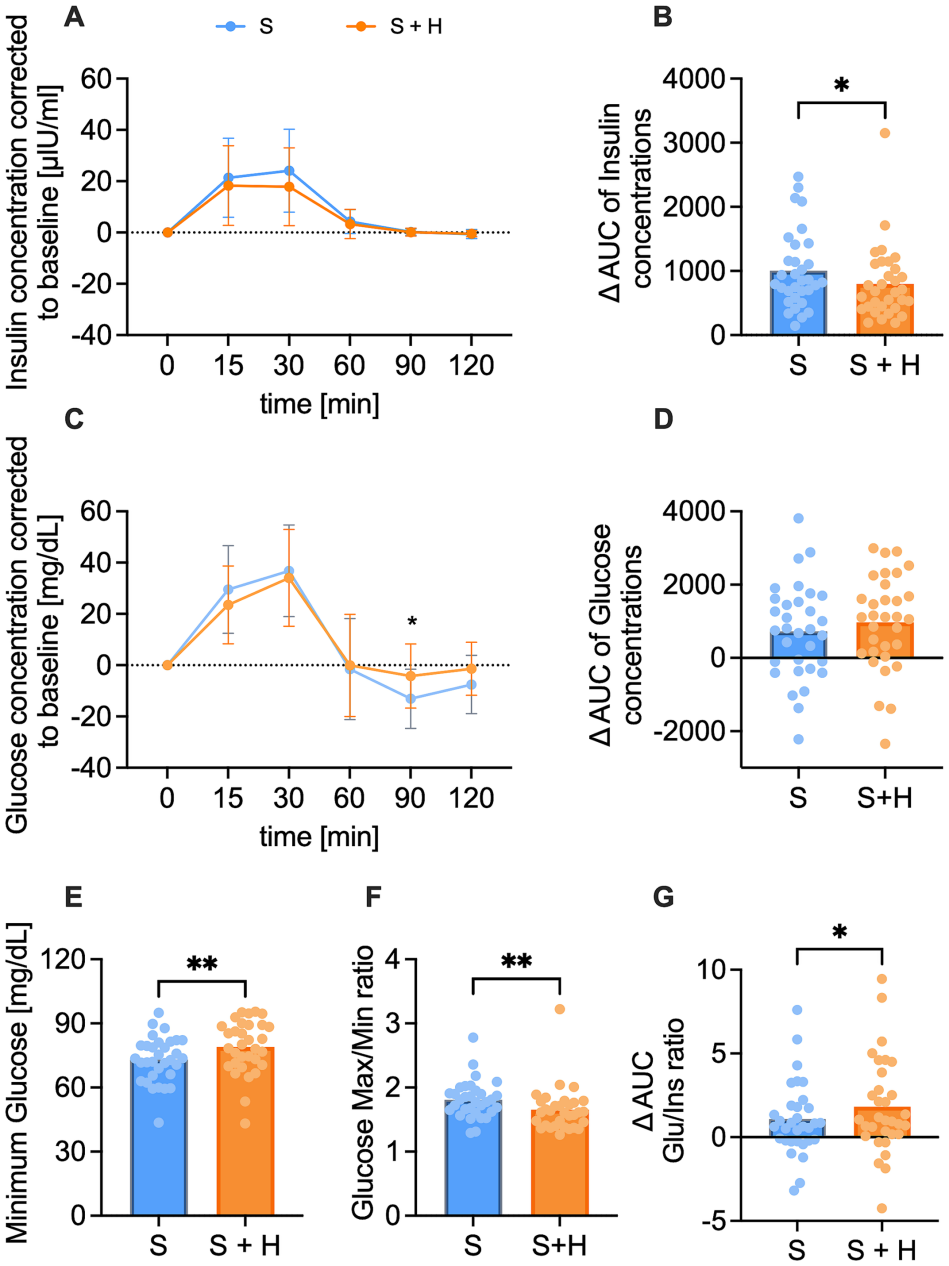
Plasma insulin [μlU/ml] and glucose [mg/dl] concentration over a time span of 120 min after consumption of the test solution S (10% sucrose) or S + H (7% sucrose with 15 mg of hesperetin). **(A)** Baseline corrected plasma concentration of insulin and **(B)** corresponding net- area under the curve (AUC) over a time span of 120 min. **(C)** Baseline corrected plasma levels of glucose and **(D)** corresponding net-AUC over a time span of 120 min. Statistical differences of plasma concentrations over time were tested by Repeated-Measures Two-way ANOVA with Šidák’s Multiple Comparison Test (*p < 0.05). Data is presented as mean ± standard deviation. Differences in AUCs were analyzed using a paired, two-tailed Student’s t-test (* *p* < 0.05). **(E)** Minimum glucose concentration over a time span of 120 min. **(F)** Ratio of maximum to minimum glucose concentration. **(G)** Ratio of mean glucose net-AUC to insulin net-AUC. Statistical differences for E-G were assessed by applying a paired Student’s t-test, two-tailed *p*-value (* *p* < 0.05, ** *p* < 0.01), (*n* = 32). Individual values (*n* = 32) for each participant are depicted by dots.

Insulin and glucose concentrations in plasma were assessed before, and 15, 30, 60, 90, and 120 min post-load. Insulin concentrations showed a regular physiological rise with a mean peak between 15 to 30 min after administration of the test solutions (Figure 2A). The net-AUC was lower after the hesperetin-spiked treatment (∆ −206.1 ± 433.5, *p* < 0.05, Figure 2B). Similar to insulin, mean glucose levels peaked between 15 and 30 min after administration of the test solutions and after 60 to 90 min either returned to the baseline level or were found below it (Figure 2C). Mean glucose concentrations 90 min after consumption of the 7%-hesperetin-containing test solution were higher than after the 10% sucrose solution (∆ 8.8 ± 3 mg/dL, *p* < 0.05). Neither the net-AUC (Figure 2D) nor the maximum glucose concentration (data not shown) differed between the two treatments. Since blood glucose concentrations after consumption of the 7% hesperetin-spiked solution did not decline to the same degree, the minimum glucose concentration was higher after the sugar-reduced solution compared to the 10% sucrose solution (∆ 5.97 ± 12.14 mg/dL, *p* < 0.05, Figure 2E). Thus, the ratio of maximum to minimum blood glucose concentrations was lower after the 7% sugar-reduced solution compared to the 10% sucrose solution (∆ −0.16 ± 0.3, *p* < 0.01, Figure 2F). In addition, we found a higher glucose/insulin ratio after the sugar reduced treatment compared to 10% sucrose (∆ 0.73 ± 2.0, *p* < 0.05, Figure 2G).

### 3.3 The desire for a sweet snack was increased after 10% sucrose solution, but not the equally sweet 7% sucrose solution

**Figure 3:**
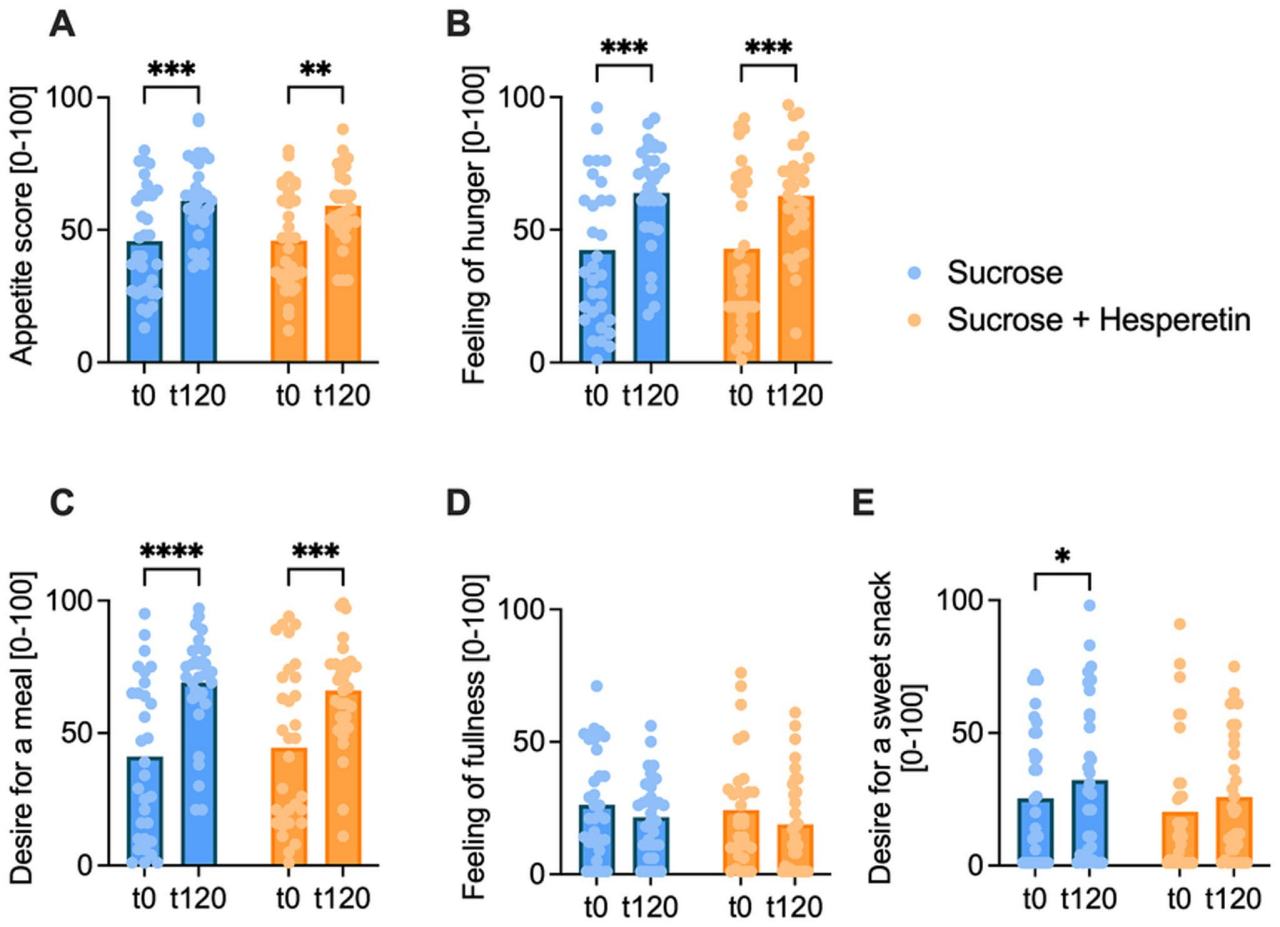
Appetite score pre- and 120 min post-load. The overall appetite score **(A)**, calculated from feeling of hunger **(B)**, desire for a meal **(C)**, feeling of fullness **(D)** and desire for a sweet snack **(E)**, was rated by the participants at baseline (t0) and 120 min after drinking of the test solution (t120) on a digital 100 mm visual analog scale. Bar charts show the mean values, dots indicate individual values, statistical differences were tested by Repeated-Measures Two-way ANOVA with Tukey’s Multiple Comparisons Test (* *p* < 0.05, ** *p* < 0.01, *** *p* < 0.001, **** *p* < 0.0001), (*n* = 32).

The mean appetite score was calculated based on individual ratings of 100 mm VAS of the four subcategories (hunger feeling, fullness feeling, desire for a meal, desire for a sweet snack) before and 120 min post-load. The mean overall appetite score increased over time after both interventions but was not different between the treatments (Figure 3A). Hunger feeling and desire for a meal increased over time after both test solutions, yet no differences between the treatments were detected (Figures 3B-C). The subjective fullness rating remained similar over time and between the interventions (Figure 3D). In contrast, the desire for a sweet snack increased after consumption of the 10% sucrose (∆ 6.8 ± 20.1 mm, *p* < 0.05), but not after the equi-sweet 7% sucrose solution with added hesperetin (∆ 5.6 ± 16.5 mm, *p* > 0.05). No differences were detected between the two treatments (∆ −1.3 ± 22.18 mm, *p* > 0.05; Figure 3E).

### 3.4 Test persons consumed less calories from the standardized *ad libitum* breakfast 2 h after the consumption of the sugar-reduced solution compared to the high sugar solution

**Figure 4:**
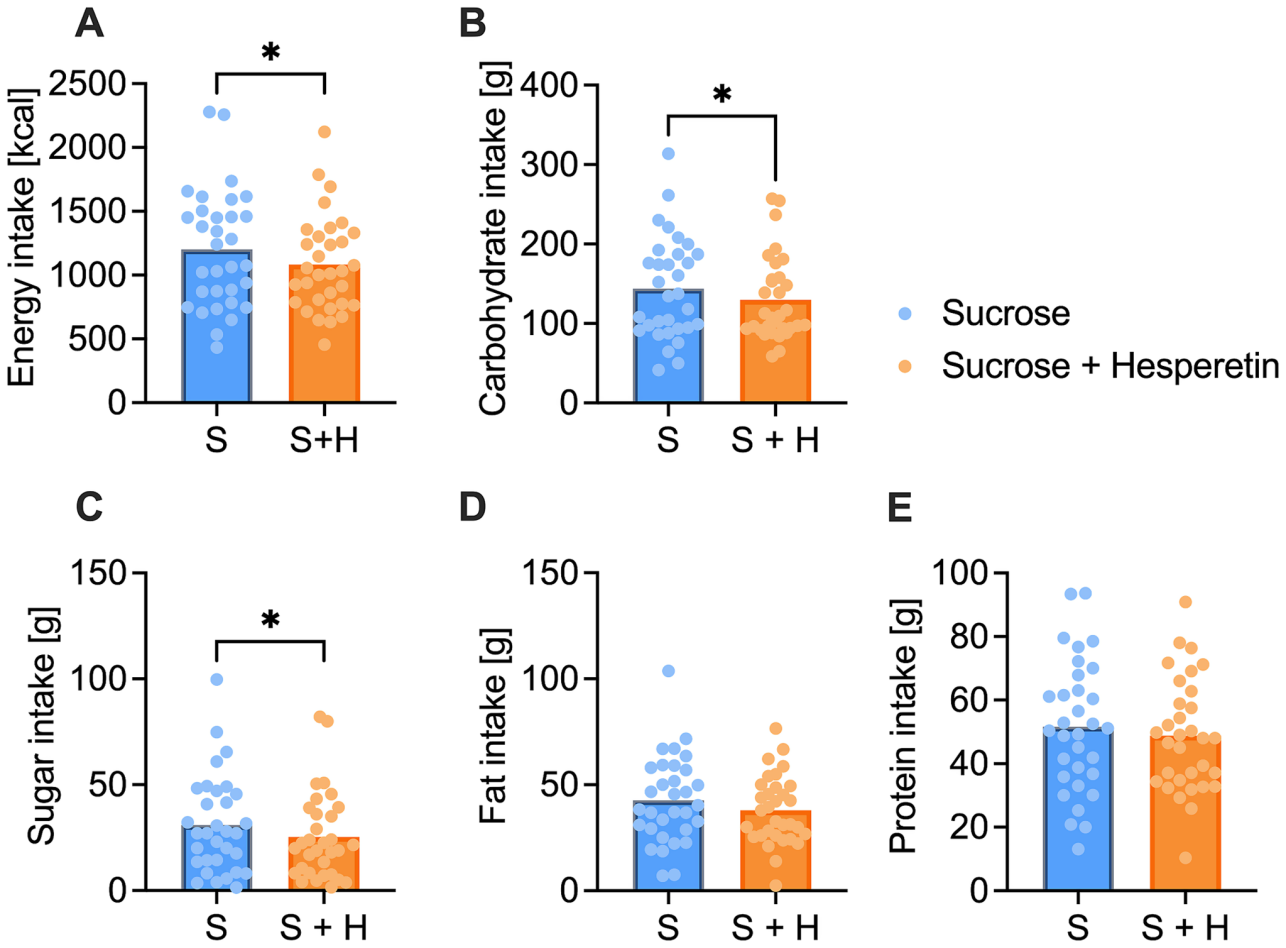
Total energy and nutrient intake of the standardized continental ad libitum breakfast. **(A)** Mean overall energy intake [kcal] **(B)**, carbohydrate intake [g] **(C)**, sugar intake [g], **(D)** fat intake [g] and **(E)** protein intake [g] 2 h after the consumption of a 10% sucrose solution (S) or a 7% sucrose solution with 15 mg of hesperetin (S + H). Statistical differences were tested by using a paired Student’s t-test, two-tailed p-value (* *p* < 0.05) (*n* = 32). The individual values of the test persons are depicted by dots.

The energy intake was assessed 2 h after the ingestion of the test solutions via a standardized *ad libitum* breakfast with a total energy content of 2,952 kcal. Participants consumed on average 9.8% less calories after administration of the 7% sucrose + hesperetin treatment compared to the 10% sucrose solution (∆ −118 ± 280 kcal, *p* < 0.05, Figure 4A). The reduced energy intake was mainly based on a decreased consumption of carbohydrates (∆ - 13.9 ± 34.3 g, *p* < 0.05, Figure 4B) and sugar (∆ −5.5 ± 14.6 g, *p* < 0.05, Figure 4C). Test persons also tended to eat less fat (∆ −4.7 ± 14.2 g, *p* = 0.07, Figure 4D), but the protein intake at the breakfast was not different between the two treatments (∆ −2.8 ± 14.5 g, *p* > 0.1, Figure 4E).

**Figure 5:**
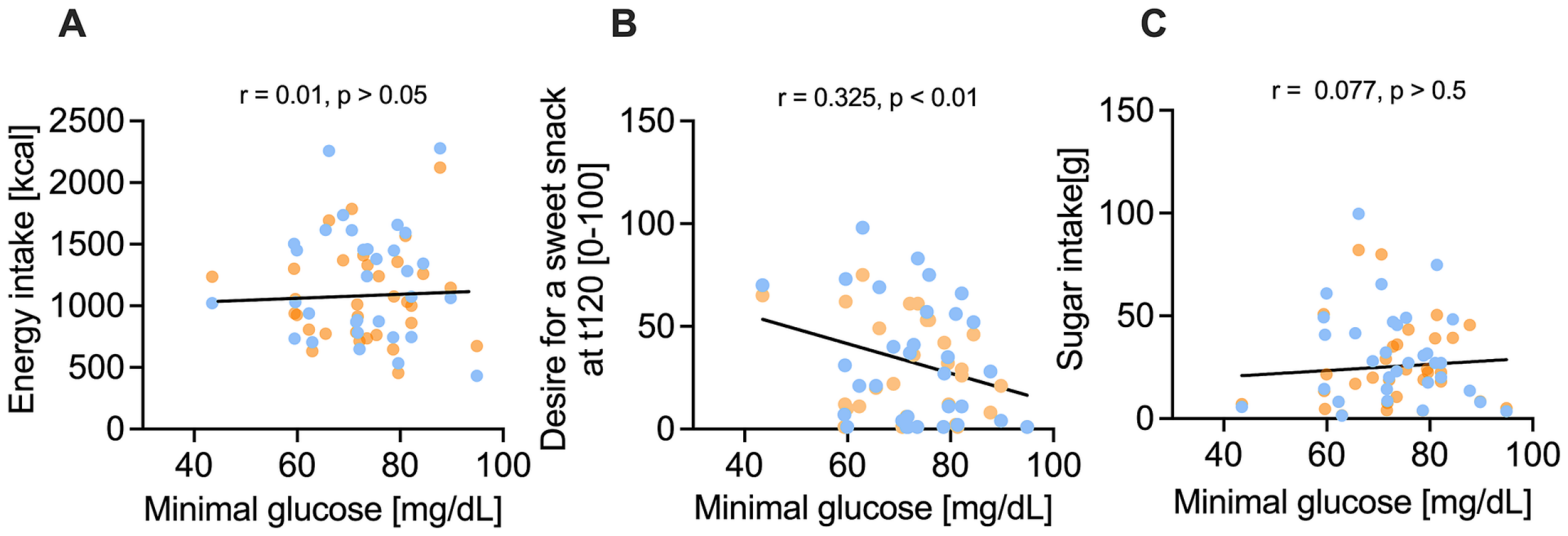
Correlation of minimum glucose concentration [mg/dl] and nutrient intake and sweet cravings after 10% sucrose solution (blue) versus 7% sucrose solution with added 50 mg/L hesperetin (orange). **(A-C)** Illustrate that nutrient intake is not correlated with the minimum plasma glucose concentration, but a lower minimum glucose concentration was associated by a significant correlation coefficient r with a higher desire for a sweet snack 120 min after consuming the test solution (t120). Statistical significance was tested by using a simple linear regression with two tailed p-value (*n* = 32).

According to our hypothesis, we next investigated whether blood glucose concentrations are associated with the appetite and subsequent energy intake. While the minimal glucose concentration after both treatments was not associated with the intake of total energy (r = 0.01, *p* > 0.05, Figure 5A) or sugar (r = 0.08, *p* > 0.05, Figure 5B), the minimal glucose concentration was inversely correlated with the craving for a sweet snack (r = −0.33, *p* < 0.01, Figure 5C). A stronger decline in blood glucose concentration after the treatments was thus associated with an increased craving for a sweet snack.

### 3.5 Reduced caloric load but equi-sweetness did only influence plasma concentrations of GIP compared to GLP-1, PYY, ghrelin and serotonin

**Figure 6:**
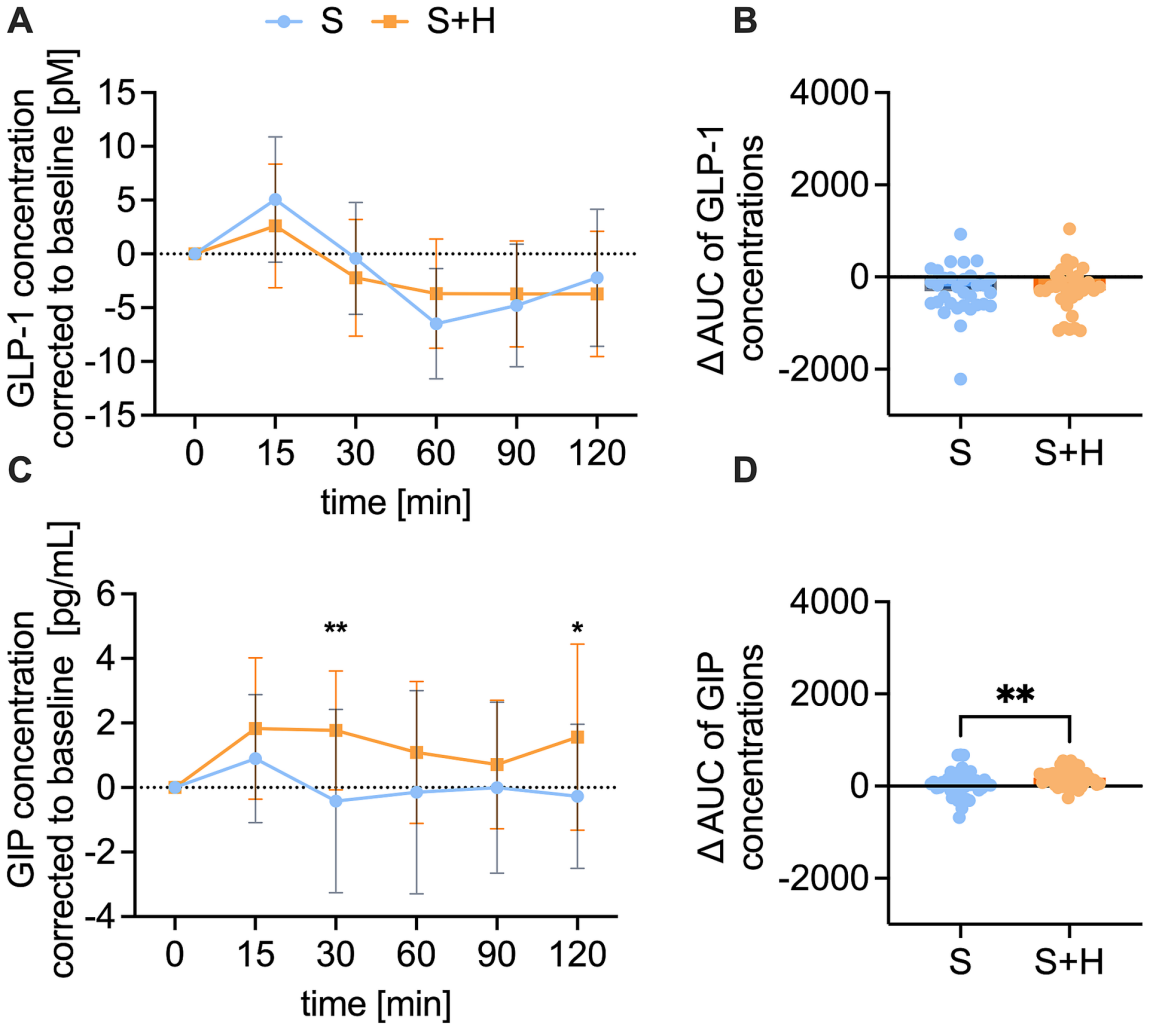
Plasma concentration of glucagon-like peptide-1 (GLP-1) [pM] and gastric inhibitory peptide (GIP) [pg/ml] after consumption of the test solution S (10% sucrose) or S + H (7% sucrose with 15 mg of hesperetin). **(A)** Baseline corrected plasma concentration of GLP-1 and **(B)** corresponding net-area under the curve (AUC) over a time span of 120 min. **(C)** Baseline corrected plasma levels of GIP and **(D)** corresponding net-AUC over a time span of 120 min. Statistical differences of time-dependent plasma concentrations were tested by Repeated-Measures Two-way ANOVA with Šidák’s Multiple Comparison Test (*p < 0.05). Values are depicted as mean ± standard deviation. Differences of AUCs were assessed using a paired, two-tailed Student’s t-test, (* *p* < 0.05, ** *p* < 0.01). The individual values (*n* = 32) of the test persons are depicted by dots.

To investigate the underlying signaling for blood glucose concentrations and energy intake, plasma concentrations of the satiety hormones GLP-1, GIP, PYY, ghrelin and serotonin were measured before, and 15, 30, 60, 90, and 120 min after administration of the test solutions. The baseline corrected, time-dependent GLP-1 levels after administration of the test solutions showed a regular time-course, with a maximum recorded after 15 min and a minimum after 60 min post-load (Figure 6A). No differences were detected in the time-dependent concentrations of GLP-1, nor the corresponding area under the curves (∆-net-AUC) between the 10% sucrose solution and the 7% sucrose solution with added hesperetin (Figure 6B). Contrary, GIP plasma concentrations were higher 30 min (∆ −2.2 ± 0.6 pg./mL, *p* < 0.01) and 120 min (∆ - 1.8 ± 0.6 pg./mL, *p* < 0.01) after consumption of the hesperetin-spiked test solution compared to the 10% sucrose solution (Figure 6C). Also, the ∆-net-AUC calculated from the GIP concentrations over time was higher after the 7% sugar solution with added hesperetin (∆ 125.4 ± 297, *p* < 0.01, Figure 6D).

The plasma concentrations of PYY, ghrelin and serotonin were not different between the two treatments (Supplementary Figure 3).

### 3.6 Hesperetin in the 7% sucrose solution led to lower enzyme activity of DPP4

**Figure 7:**
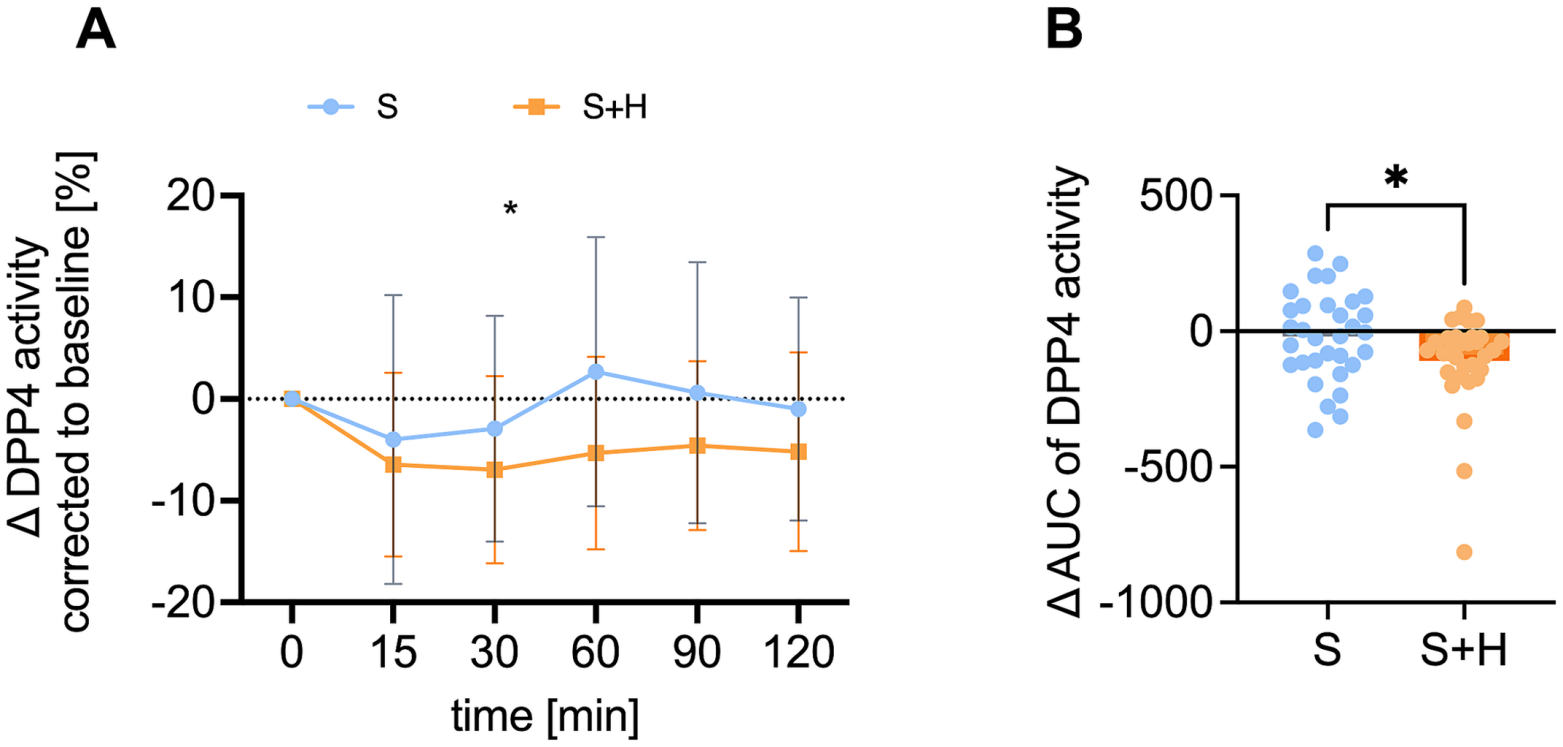
Plasma activity of dipeptidyl-peptidase 4 (DPP4) [%] after administration of the test solution S (10% sucrose) or S + H (7% sucrose with 15 mg of hesperetin). **(A)** Baseline corrected DPP4 activity in plasma over a time span of 2 h. Statistical differences were tested by Repeated-Measures Two-way ANOVA with Šidák’s Multiple Comparison Test (*p < 0.05), (*n* = 32). Values are depicted as mean ± standard deviation. **(B)** Mean plasma net-area under the curve (AUC) of DPP4. Statistical differences were tested with paired two-tailed Student’s t-test, (* *p* < 0.05), (*n* = 32). The individual values of the test persons are depicted by dots.

To examine, whether the higher GIP levels after consumption of the 7% sucrose solution with hesperetin might be the result of a reduced DPP4 activity, plasma activity of DPP4 was measured at baseline and 15, 30, 60, 90 and 120 min after consumption of the test solutions. The baseline corrected DPP4 activity was significantly lower 60 min after administering the hesperetin-spiked 7% sucrose solution opposed to the 10% sucrose solution (∆ - 8.0 ± 2.8%, *p* < 0.05) (Figure 7A). Furthermore, after the 7% sucrose solution with hesperetin, a lower ∆-net-AUC (∆ - 89.8 ± 221.9, *p* < 0.01, Figure 7B) was detected.

## 4 Discussion

Building on previous findings (16, 18, 35), the present study investigated whether reducing the sugar content of a sucrose solution while maintaining its perceived sweetness intensity through the addition of hesperetin attenuates postprandial blood glucose fluctuations, potentially leading to decreased appetite and a consequent reduction in energy intake. As the fundamental base for elucidating this hypothesis we first confirmed that the sensorially untrained participants of the study rated the two test solutions, a 10% sucrose solution and a 7% sucrose solution with 50 mg/L hesperetin as equally sweet. In line with our hypothesis, the participants’ blood glucose concentrations exhibited a less pronounced decline 90 min after consumption of the 7% sucrose supplemented with hesperetin compared to the 10% sucrose. While a previous study detected no blood glucose levels below baseline after administration of 29 g of sucrose (34), we discovered that the blood glucose concentrations of the participants declined below the baseline concentrations after both treatments (30 g vs. 21 g sucrose + 15 mg hesperetin) between 90 and 120 min, although no hypoglycemic blood sugar concentrations occurred.

Since large fluctuations in blood glucose concentrations have been associated with increased cravings and higher energy intake (34, 37), we hypothesized that reduced glycemic fluctuations contributed to the reduced energy intake.

Overall, we did not detect differences in fullness ratings over time, supporting the hypothesis that sugar sweetened beverages alone may have limited capacity to stimulate robust satiation mechanisms compared to solid food due to their high content of easily metabolized carbohydrates (38). Even though we discovered differences in the satiety hormones insulin and GIP, this did not lead to changes in actual fullness feelings. Similarly, no treatment-specific differences were found for overall appetite score or for the individual hunger-, satiety feeling or the desire for a meal or a sweet snack. One possible explanation is that the sucrose difference of 9 g between the test solutions may not been large enough to elicit measurable effects. Yet our findings align well with Markus and Rogers (34), who also reported no treatment-specific differences in appetite score 165 min after administration of a low (29 g) or high (80 g) sugar-containing beverage. However, our findings revealed that the desire for a sweet snack increased over time following consumption of the 10% sucrose solution but remained stable after the sucrose-reduced solution containing hesperetin. This suggests that participants experienced lower cravings for sweet foods after the 7% sucrose solution with hesperetin, which is also reflected in the nutrient intake data from the *ad libitum* breakfast. Test persons consumed specifically less carbohydrates and sugar after the hesperetin-spiked solution compared to the 10% sucrose solution, resulting in an overall reduced total energy intake by 10%. In addition, whereas the intake of total energy or sugar at the *ad libitum* breakfast was not related to blood glucose fluctuations, participants indicated less desire for a sweet snack when blood glucose levels did not drop as drastically. This supports our hypothesis of attenuated blood glucose fluctuations leading to decreased total energy intake.

To investigate a potential mechanism behind the differences in glycemic fluctuations and the decreased energy intake, the release of incretin and satiety hormones was investigated. An increased secretion of GLP-1, GIP, PYY, ghrelin and serotonin is a physiological consequence to taste stimuli and is modulating key components of digestion, like gastric emptying, nutrient absorption and metabolic processes (39). This contributes to appetite and thus influences food intake (40). In a fasted state, plasma concentrations of the incretin hormones GLP-1 and GIP, but also PYY and serotonin are low and start rising for several hours after food intake (40–42). The secretion of the incretin hormones GLP-1 and GIP post-load further plays a role in the stimulation of insulin release to ameliorate blood glucose levels (43). The increasing incretin hormone concentrations 15 to 30 min after ingestion of the test solutions in our study confirm that the secretion is stimulated upon carbohydrate uptake in the intestine (44). Therefore, we hypothesized that incretin levels would be higher after treatment with 10% sucrose than 7% sucrose with added hesperetin. However, we did not detect differences in GLP-1, PYY and serotonin levels between treatments. This supports that the sweetness level and the sweet taste receptor activation are important for the release of those hormones. Previous human studies also provided data in line with a role of the sweet taste receptor for the secretion of those hormones. Findings of Grüneis et al. (16) and Schweiger et al. (18) showed that the addition of 18 mg of the sweet taste inhibitor and sweet receptor antagonist lactisole decreased sucrose-induced increase in GLP-1 (16) and serotonin (18) concentrations. In addition, a decreased secretion of GPL-1 and PYY when blocking the sweet taste receptor with lactisole has been reported (4, 45). To summarize, the results of the present study support the idea of a regulatory role of the sweet taste receptor for the secretion of GLP-1, PYY, and serotonin. However, it cannot be excluded that a 30% reduction of the sugar content was not high enough to detect differences in the release of those hormones. In addition, the differences in energy intake cannot be explained by those hormones.

In contrast to GLP-1, GIP plasma concentrations were higher following the sucrose-reduced solution compared to 10% sucrose. Incretin hormones are degraded by the enzyme DPP4. A previous study proposed that phenolic compounds derived from citrus fruits, including hesperetin, are able to inhibit enzymatic activity of DPP4 (46). Also in our study, the DPP4 plasma activity was lower after consumption of the hesperetin-spiked solution compared to the 10% sucrose solution. It is thus conceivable that the reduced DPP4 activity resulted in the higher GIP concentrations after consumption of the 7% sucrose solution with hesperetin. However, it does not explain why this was only found for GIP and not for the second analyzed incretin hormone, GLP-1. One reason could be that GLP-1 gets additionally degraded in the liver, so that only 10–15% enter the systemic cycle (47). Another study supports that even less than 5% of intact GLP-1 enters the systemic cycle (48). Therefore, it is possible that GLP-1 is rapidly degraded prior to the onset of any DPP4 inhibitory activity exerted by hesperetin.

This study has potential limitations. Surprisingly, despite the differences in caloric load (30 g vs. 21 g sucrose), maximum blood glucose concentrations did not differ after administration of the two test solutions. This could be due to the equal activation of the sweet taste receptor compensating for the reduced sugar content, as previously hypothesized (49), although not consistently supported *in vivo* (50, 51). However, we cannot rule out the possibility that hesperetin itself influenced blood glucose regulation, since various *in vitro* and animal studies indicated beneficial effects of hesperetin on glycemic regulations For instance, it has been demonstrated that hesperetin decreased basal glucose uptake in monocytic U937 cells (36) and MDA-MB-231 breast cancer cells (52) and molecular docking and virtual screening studies have found hesperetin to inhibit the α-glucosidase and α-amylase (53). Previous studies in rats have shown that both hesperidin and its aglycone hesperetin improve blood glucose levels. However, the doses used ranged from 40 to 100 mg/kg body weight and were administered daily for several weeks (54, 55). Therefore, these findings cannot be extrapolated to our human study population, receiving just 15 mg of the substance once. Future studies are therefore warranted to elucidate the impact of hesperetin on human blood glucose regulation, ideally employing appropriate control conditions to isolate its effects.

Furthermore, no females were included in this study due to fluctuations in blood sugar regulation caused by menstrual cycle described in previous studies (31). Thus, sex-specific effects on postprandial glucose response and secretion of satiety markers cannot be excluded and needs be addressed in larger studies. Our findings can further not be extrapolated to patients with impaired glucose regulation, since only healthy individuals participated in this study. Moreover, postprandial glucose response is additionally influenced by numerous psychological variables, such as stress, sleep deprivation and dehydration (56, 57). Even though we advised study participants to arrive in a relaxed state and have similar night-meals the day bevor and used a controlled laboratory environment to minimize environmental stress, and excluded participants with any acute illness or pain, this work did not formally assess stress levels or other psychological factors that could have interfered with glucose levels. Also, we cannot rule out the involvement of additional mechanisms, such as pathways activated by extra-oral bitter taste receptors, since hesperetin exhibited bitter-masking effects (23). These receptors have been demonstrated to play a role in postprandial energy homeostasis by inducing GLP-1 secretion, stimulating gastrointestinal motility and enhancing satiety signaling, as summarized by Harmon et al. (58). Finally, long-term intervention studies are warranted to determine whether the observed reduction in energy intake is sustained over an extended period. Further studies are required to assess the safety of hesperetin with repeated exposure and investigate potential beneficial or adverse compound-specific effects of hesperetin in long-term interventions. Future long-term investigations should incorporate comprehensive metabolic assessment, including insulin resistance measures such as Homeostatic Model Assessment (HOMA), and evaluate potential impacts on gut microbiome composition and function. Additionally, our study design cannot determine the underlying mechanisms driving differences in nutrient intake patterns. Food intake behavior is influenced by complex interactions of physiological, psychological, and environmental factors, as well as individual food preferences that extend beyond the appetite-regulating hormones and subjective appetite ratings we measured. Therefore, future studies should explore these mechanistic relationships more thoroughly and incorporate food preference assessments to better tailor interventions and enhance the reliability of food intake measurements. However, this study was the first to compare the effects of a sucrose solution with a combined solution of sucrose with a flavor modulator to explore the involvement of the sweet taste receptor on blood glucose regulation and energy intake.

In conclusion, we demonstrated that reducing the sugar content of a sucrose solution in dietary relevant concentrations, while maintaining the perceived sweet taste intensity using the flavoring substance hesperetin, attenuated postprandial blood glucose fluctuations. This effect was associated with reduced cravings for sweet snacks and ultimately resulted in decreased total energy intake during a standardized breakfast in healthy male subjects. This is an important finding in the context of ongoing efforts to reduce dietary sugar intake and implement effective sugar reduction strategies.

## Data Availability

The original contributions presented in the study are included in the article/Supplementary material, further inquiries can be directed to the corresponding author.
