# Supplementary material for: Sucrose reduction with maintained sweetness level lowers glycemic fluctuations and energy intake in healthy males
Source: Front Nutr. 2025 Nov 3;12:1682297. doi: 10.3389/fnut.2025.1682297 (PMC12621453; doi:10.3389/fnut.2025.1682297)
Supplement: Supplementary file 1 [file Table_1.DOCX]

Supplementary Material

Marlies Gaider^1,2,3^, Isabella Kimmeswenger^2,4^, Jana Schmidt^3^, Cynthia Thines^1^, Anni Wu^1^, Teresa K. Stoffl^1^, Petra Rust^5^, Jakob P. Ley^6^, Gerhard E. Krammer^6^, Veronika Somoza^4^, Barbara Lieder^1,3,*^

^1^Christian Doppler Laboratory for Taste Research, Faculty of Chemistry, University of Vienna, Vienna, Austria ^2^Vienna Doctoral School of Chemistry (DoSChem), University of Vienna, Vienna, Austria ^3^Institute of Clinical Nutrition, University of Hohenheim, Stuttgart, Germany ^4^Institute of Physiological Chemistry, Faculty of Chemistry, University of Vienna, Vienna, Austria ^5^Department of Nutritional Science, Faculty of Life Sciences, University of Vienna, Vienna, Austria ^6^Symrise AG, Holzminden, Germany

*** Correspondence:**Barbara Lieder
Barbara.Lieder@uni-hohenheim.de

**Supplementary Figure 1.** Sweetness intensity [cm] of a 10 % sucrose solution vs a 7% sucrose solution with added 50 mg/L hesperetin in a pre-tasting with a sensorially trained panel (n=25) **(**A); on the study days with untrained study participants (n=32) (B), Statistical differences were tested by using a paired two-tailed Students’s t-test. Values are depicted as mean + SD.

**Supplementary Figure 2.** Distribution of sweet taste detection threshold [g/L] (A), body weight [kg] (B), BMI [kg/m^2^] (C) and body fat [%] (D) among study population (n = 32).

**Supplementary Figure 3.** Plasma concentrations of PYY [pg/mL], Ghrelin [pg/mL] and Serotonin [ng/mL] after consumption of the test solution S (10 % sucrose) or S+H (7 % sucrose with 15 mg of hesperetin. (A, C, E) Baseline corrected plasma concentration of PYY, ghrelin and serotonin over a time span of 120 min, respectively. Statistical differences were tested by Repeated-Measures Two-way ANOVA with Šidák´s Multiple Comparison Test (*p<0.05), (n=32). Values are depicted as mean ± SD. The figures (B, D, F) depict the mean plasma net-AUC of PYY, ghrelin and serotonin Statistical differences were tested by applying a paired, two-tailed Student’s t-test, (* p<0.05), (n=32). The individual values of the test persons are depicted by dots.

Supplementary Table 1. Nutritional composition of food items served at the standardized breakfast

Omnivore option:

| Food item | Carbohydrates  [g/100g] | Fat  [g/100g] | Protein  [g/100g] | Calories  [kcal/100g] |
| --- | --- | --- | --- | --- |
| Bread roll | 49 | 4.2 | 8.5 | 274 |
| Mixed-flour bread | 41 | 3.2 | 8.8 | 240 |
| Cheese (Gouda-type) | 0 | 45 | 24 | 348 |
| Ham | 0.5 | 8.5 | 18 | 151 |
| Butter | 0.6 | 82 | 0.7 | 743 |
| Strawberry jam | 55 | 0 | 0.6 | 225 |
| Honey | 75 | 0 | 0.4 | 302 |
| Coffee creamer | 4 | 10 | 3.1 | 118 |
| White sugar | 100 | 0 | 0 | 400 |
| Dairy Yoghurt with mixed-berries flavor | 11 | 2.6 | 3.9 | 84 |

Plant-based option

| Food item | | Carbohydrates  [g/100g] | | Fat  [g/100g] | | Protein  [g/100g] | Calories  [kcal/100g] | |
| --- | --- | --- | --- | --- | --- | --- | --- | --- |
| Bread roll | 45 | 1.6 | | 8.1 | | | 233 |  |
| Mixed-flour bread | 47 | 0.5 | | 5.8 | | | 226 |  |
| Vegan cheese | 22 | 20 | | 1.5 | | | 274 |  |
| Vegan ham | 7.3 | 4.9 | | 3.6 | | | 94 |  |
| Plant-based butter | 0.5 | 79 | | 0.5 | | | 713 |  |
| Strawberry jam | 55 | 0 | | 0.6 | | | 225 |  |
| Agave syrup | 75 | 0 | | 0.4 | | | 302 |  |
| Oat-based cooking cream | 6.2 | 13 | | 1 | | | 148 |  |
| White sugar | 100 | 0 | | 0 | | | 400 |  |
| Soy yoghurt with blueberry flavor | 8.2 | 2.2 | | 3.7 | | | 70 |  |
